# Effect of acupuncture on insomnia in menopausal women: a study protocol for a randomized controlled trial

**DOI:** 10.1186/s13063-019-3374-8

**Published:** 2019-05-30

**Authors:** Shanshan Li, Ping Yin, Xuan Yin, Anna Bogachko, Tingting Liang, Lixing Lao, Shifen Xu

**Affiliations:** 1 Shanghai Municipal Hospital of Traditional Chinese Medicine, Shanghai University of Traditional Chinese Medicine, Shanghai, 200071 China; 2 School of Chinese Medicine, University of Hong Kong, Hong Kong, China; 3 University of Maryland School of Medicine, Baltimore, MD 21201 USA

**Keywords:** Menopause, Insomnia, Acupuncture, Clinical trial

## Abstract

**Background:**

The National Institutes of Health estimates the prevalence of insomnia in menopausal women at 40–50%. Some studies have shown that acupuncture might be effective in treating primary insomnia and insomnia related to depression and stroke. Although there are some programs supporting insomnia during the menopausal transition, there are few randomized controlled trials (RCT) to provide evidence regarding their effectiveness. We design a RCT of suitable sample size to verify the effectiveness of acupuncture in patients with insomnia during the menopausal transition and to form an optimized acupuncture treatment protocol.

**Method/Design:**

In this randomized, single-site, single-blind, placebo-controlled trial, 84 eligible patients will be recruited and randomly assigned to either the acupuncture group (*n* = 42) or the sham control group (*n* = 42) in a 1:1 ratio. Participants will receive a total of 18 treatment sessions for eight consecutive weeks. Treatments will be given three times per week in the first four weeks, twice a week for the next two weeks, and finally once weekly for the final two weeks. Treatment will utilize eight main acupoints (GV20, GV24, GV29, RN6, RN4, SP6, HT7, EX-HN22) and extra two acupoints based on syndrome differentiation. The primary outcome will be assessed using the Pittsburgh Sleep Quality Index (PSQI). The secondary outcomes will be measured by sleep parameters recorded in the Actigraphy (SE, TST, SA), Insomnia Severity Index (ISI), Self-Rating Anxiety Scale (SAS), Self-Rating Depression Scale (SDS), and Menopause Quality of Life (Men-QOL). The primary outcomes will be assessed at baseline, week 4, week 8, and the first and third month after the end of treatment.

**Discussion:**

If the results confirm that acupuncture is effective and safe for the treatment on insomnia in menopausal women, this positive outcome could provide evidence for clinical application.

**Trial registration:**

Chinese Clinical Trial Registry, ChiCTR1800018645. Registered on 10 January 2018.

## Background

The menopausal transition (MT) is often accompanied by a myriad of symptoms, the most common being insomnia [1–3]. Insomnia is characterized by having difficulty falling asleep, frequent nocturnal waking with difficulty falling back asleep [4], and unexplained pain. A study conducted by the National Institutes of Health estimates the prevalence of insomnia in menopausal women at 40–50% [5–8]. Women who experience poor sleep quality during the MT are more likely to have critical problems [9] such as depression, anxiety, memory and cognitive deprivation, cardiovascular disease, and reduced quality of life [3, 4]. Still, there are few effective therapies available for treating sleep problems during the MT. Yoga, therapeutic massage, and exercise may be helpful for sleep disorders, but they have not yet been proven as an effective independent treatment [10]. Hormone therapy (HT) is currently the most effective treatment but is also associated with a number of risks [11, 12]. HT can reduce hot flashes at night; some studies have reported that it can reduce sleep disorders [13]. However, increasing numbers of women are refusing HT due to the risks (myocardial infarction, stroke, venous thromboembolism, and breast cancer) outlined by trials published in the Women’s Health Initiative [14]. Moreover, these trails reported that HT has a weaker effect on insomnia than previously thought [15] .

Acupuncture, an important part of traditional Chinese medicine (TCM), has been used for thousands of years in China to treat various diseases including sleep disorders [5]. Many studies have confirmed the efficacy of acupuncture for primary insomnia and related depressive disorders [16–18]. Based on results of the efficacy of acupuncture for depression-related insomnia [19] and insomnia following stroke [20], we believe that acupuncture might be an effective treatment for insomnia during the MT. However, effects of acupuncture on sleep disorders remain uncertain because of flawed research methodology, such as improper sample size, treatment times, and follow-up problems [21].

The main objective of this study is to determine whether acupuncture is effective as compared to a sham acupuncture control on insomnia in menopausal women. The findings of this trial will provide useful information in forming an optimal acupuncture treatment protocol.

## Methods/Design

### Hypotheses

Acupuncture treatment helps menopausal transition insomnia patients improve their sleep quality and other related symptoms.

### Objectives

1. To compare the differences in improvement of insomnia, assessed by the Pittsburgh Sleep Quality Index (PSQI) and Insomnia Severity Index (ISI), between the intervention group and control group.
2. To compare the differences in improvement of mood, measured by the Self-Rating Anxiety Scale (SAS) and Self-Rating Depression Scale (SDS), between the intervention group and control group.
3. To compare the differences in improvement of Menopause Quality of Life (MenQOL), between the intervention group and control group.

### Design

**Fig. 1:**
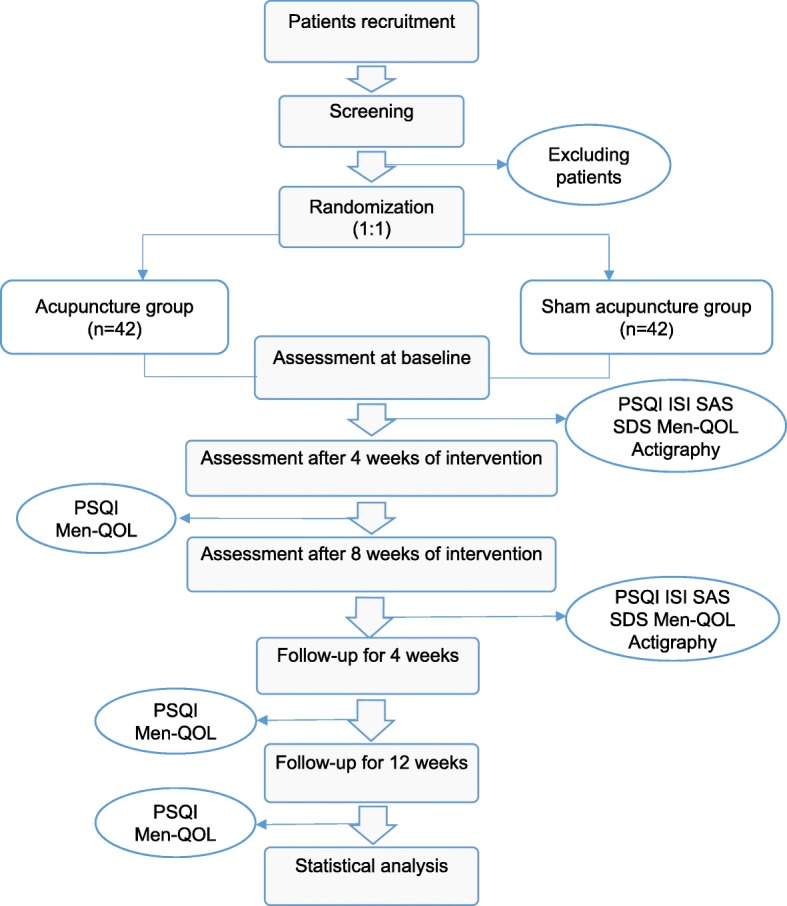
Flowchart of the trial

This is a single-site, single-blind, randomized, placebo-controlled clinical trial that will be carried out in the Shanghai Municipal Hospital of Traditional Chinese Medicine. Eligible patients will be randomly divided into the acupuncture group and the sham acupuncture group in a 1:1 allocation ratio. All participants will sign the informed consent before proceeding with the trial. The flow chart of the study process is as follows in Fig. 1. Timing of treatment assessments and data collection are as follows in Table 1.

**Table 1:** Timing of treatment assessments and data collection

	Study period					
	Enrolment	Baseline	Treatment phase		Follow-up phase	
Timepoint^a^	–1 week	0 weeks	4 weeks	8 weeks	4 weeks	12 weeks
Enrolment						
Eligibility screen	X					
Informed consent	X					
Medical history	X					
Allocation		X				
Interventions						
Acupuncture		X	X	X		
Sham acupuncture		X	X	X		
Assessments						
Primary outcome						
PSQI		X	X	X	X	X
Secondary outcomes						
ISI		X		X		
Actigraphy		X		X		
SDS		X		X		
SAS		X		X		
Men-QoL		X	X	X	X	X
Others						
Estazolam dose		X	X	X	X	X
Adverse events		X	X	X	X	X
Patients’ satisfaction						X
Success of blinding						X

### Recruitment

Participants of the study will be recruited through the outpatient clinic, hospital-based Wechat advertising, and posters in Shanghai Municipal Hospital of Traditional Chinese Medicine in Shanghai, China. Those who are interested in participating in the study will be screened either via telephone or on-site. They will receive eight weeks of acupuncture treatment or sham acupuncture and a series of assessments on sleep disorders free of charge. During the study, participants will be required to wear a wrist Actigraphy to record their sleep quality. The participants who meet the inclusion criteria will be informed of the research details and asked to sign the informed consent before the treatment commence. The research assistant will obtain informed consent from participants.

### Inclusion criteria

Participants with the following conditions will be included:

1. Menopausal women aged 45~60 years;
2. Participants who meet the diagnostic criteria of TCM in accordance with the above perimenopausal and insomnia [22];
3. Participants in line with the international classification of sleep disorders (ICSD-3) for the diagnosis of insomnia;
4. Participants whose PSQI score is > 5;
5. Participants who voluntarily agree to join the study and to sign the written informed consent form for the randomized controlled trial (RCT).

### Exclusion criteria

Participants with the following conditions will be excluded:

1. Participants who have severe mental illness such as depression, anxiety, schizophrenia, etc.;
2. Participants with serious heart, brain, kidney, or liver disease;
3. Participants with secondary insomnia caused by systemic diseases such as pain, fever, cough, surgery, and external environment disturbances;
4. Participants who have taken hormones for ≥ 6 months;
5. Participants who are pregnant or currently lactating.

### Intervention

Participants in each group will receive 18 real or sham treatments within an eight-week period. In each group, they will receive either acupuncture or sham acupuncture treatment three times per week for the first four weeks, twice per week for the next two weeks, and once per week for the last two weeks. Each treatment will last for 30 min. Each patient will then be placed in a separate quiet space and lying supine. The acupuncturists will be registered practitioners with > 3 years of experience in acupuncture practice. In order to increase the patients’ compliance, they will accept the intervention and assessment by reservation and they will receive some financial subsidies (200 RMB) after the follow-up assessments.

In the treatment group, acupuncture needles will be standard stainless steel, sterile, and disposable (0.25 × 40 mm and 0.30 × 40 mm in length; Jia Jian, China). The control group will use the Streitberger Placebo-needle at the same acupoints. The treatment methods of acupuncture and acupoints are shown in Table 2.

**Table 2:** Details of intervention

	Intervention group	Control group
Main acupoints	GV20, GV24, GV29, RN6, RN4, SP6, HT7, EX-HN22	GV20, GV24, GV29, RN6, RN4, SP6, HT7, EX-HN22
Added acupoints	Kidney yang deficiency GV4, BL23	Kidney yang deficiency GV4, BL23
	Kidney yin deficiency KI3, KI7	Kidney yin deficiency KI3, KI7
Depth of insertion	GV20, GV24, GV29, RN6, RN4, EX-HN22 10 mm	No insertion
	HT7, SP6, GV4, BL23, KI7, KI3 15 mm	
Needle type	Steel needle (Wuxi Jiajian Medical Co. Ltd., Wuxi, China)	Blunt-tip needle (Streitberger Placebo-needle)
Needle sensation	With de-qi sensation	Without de-qi sensation
Electric stimulation	Needle on GV20, GV29 connected to G6805–2 Multi-Purpose Health Device (Shanghai Medical Instruments High-Techno, China) with electric pulse at a frequency of 2.5 Hz and an intensity of 45 mA	Needle on GV20, GV29, connected to G6805–2 Multi-Purpose Health Device (Shanghai Medical Instruments High-Techno, China) without electric pulse
Frequency and duration	Three times per week for the first four weeks	Three times per week for the first four weeks
	Twice per week for the next two weeks	Twice per week for the next two weeks
	Once per week for the final two weeks	Once per week for the final two weeks

### The acupuncture group

Participants in this group will receive acupuncture treatment during the first eight weeks. According to our in-depth research on insomnia and acupuncture experience, Baihui (GV20), Shenting (GV24), Yintang (GV29), Qihai (RN6), Guanyuan (RN4) and bilateral Anmian (EX-HN22), Sanyinjiao (SP6), and Shenmen (HT7) will be used as main acupoints [17]. Acupuncturists will be permitted to add acupuncture points based on TCM syndrome differentiation [23]. According to our own clinical practice, potentially relevant extraneous points include Mingmen (GV4) and Shenshu (BL23) for kidney yang deficiency and TaiXi (KI3) and Fuliu (KI7) for kidney yin deficiency. The needles will be inserted into the skin to the depth of 10–30 mm and manipulated manually (including lifting, thrusting, and rotating) until the patient reports needling sensations (Deqi sensation). The needles on GV20 and GV29 will be connected to a G6805–2 Multi-Purpose Health Device (Huayi Company), using continuous wave type, frequency at 2.5 HZ, and intensity of 45 mA. Needles will be retained for 30 min before removal.

### The control group

Participants in this group will receive sham acupuncture treatment. We will use a non-invasive placebo control, the Streitberger Placebo-needle. Acupoints are the same as with acupuncture group, without insertion. An electroacupuncture apparatus (G6805–2 Multi-Purpose Health Device) will be set beside the patients and connected to the GV20 and GV29, without electrical pulse. Needles will be also retained for 30 min before removal.

### Outcome measures

We will assess the primary outcome at baseline, week 4, week 8, after treatment, and with follow-ups at week 4 and week 12 after the end of treatment. Secondary outcomes will be assessed at baseline and week 8.

### Primary outcome

#### Pittsburgh Sleep Quality Index (PSQI)

The PSQI is a self-rated questionnaire used to measure general sleep quality. It comprises 19 self-rated items and five other-rated items [20]. It assesses sleep based on seven domains in the past month: sleep duration; sleep disorders; sleep-onset latency; daytime dysfunction; sleep efficiency; use of medications to sleep; and overall sleep quality [24]. Each domain is rated 0–3; the accumulated scores of the seven domains constitute the total score of the PSQI (0–21). A total score of > 5 indicates a poor quality of sleep [25] .

### Secondary outcomes

#### Insomnia Severity Index (ISI)

The ISI is designed to assess the nighttime and daytime symptoms of insomnia. It includes seven items rated on a scale of 0–4 points; the total score is in the range of 0–28 [26]. All self-reported questionnaires are conducted in Chinese. A higher score indicates a more severe insomnia. ISI classification: clinical insomnia (score 0–7), mild insomnia (score 8–14), moderate insomnia (score 15–21), and severe insomnia (score 22–28).

#### Actigraphy assessments

The wActiSleep-BT actigraph (Actigraph LLC, Pensacola, FL, USA), will be worn on the patients’ wrist, which can record the sleep quality by noting sleep awakenings (SA), total sleep time (TST), sleep onset, sleep latency as well as the efficiency of sleep. The analysis of sleep condition and sleep quality will be performed by the software ActiLife6 (Version 6.8.1, Actigraph LLC) [17, 20].

#### Self-Rating Anxiety Scale (SAS)

We will use SAS to assess the degree of anxiety in patients. The questionnaire includes 20 items, as follows: 15 negative and five positive descriptions, the opposite of the score [27]. The total score of the 20 items is > 40 points, indicating the state of anxiety. A higher score indicates more severe anxiety.

#### Self-Rating Depression Scale (SDS)

The SDS is a self-rated scale for evaluating the severity of depression [28]. The main statistical index consists of 20 questions. We first calculate the standard score by ranking the participant’s responses to 20 questions. From this figure, the total score determined as is 1.25 times the standard score, making use of the integral part [17]. The demarcation point of depression is 50 points, the higher the score, the more significant the depression tendency.

#### Menopause Quality of Life (MenQOL)

MenQOL is a questionnaire used to assess the quality of life in menopause, including 29 items, which are divided into four domains: Vasomotor (Items 1–3); Psychosocial (Items 4–10); Physical (Items 11–26); and Sexual (Items 27–29). For analyses, we convert the item scores to a score in the range of 1–8 (NO, 0–6). Each domain mean is in the range of 1–8. The overall questionnaire score is the mean of the domain means. A higher score indicates a worse quality of life [29].

#### Estazolam dose

Patients will be allowed to take Estazolam (0.5–2.0 mg) when they have difficulty in falling asleep for > 3 consecutive days during the trial. They will be asked to record the dose and the time of taking Estazolam on the case report form (CRF).

#### Sample size

The sample size calculation was based on the change of PSQI scores. Systematic review [21] shows that at least a 2.70-point difference in PSQI scores between acupuncture and sham acupuncture has clinical significance. We expected that acupuncture would outperform the sham acupuncture by 3 points; therefore, a sample size of 35 participants should be recruited in each group. Considering a dropout rate of ~ 20%, each group will take 42 cases. Therefore, a total of 84 participants should be recruited for this RCT.

$$\mathrm{N}=2\ast \kern0.5em {\left[\frac{\left({U}_{\upalpha}+{U}_{\upbeta}\right)\upsigma}{\updelta}\right]}^2;\kern0.5em \left(\upalpha =0.05,\upbeta =0.2\right)$$

#### Randomization and allocation concealment

We will use the block randomization method. We plan to use SPSS version 23.0 software to generate a random number table. The participants who meet the criteria will be randomly assigned to acupuncture group or control group with 1:1 ratio. The random allocation sequence will be generated in a block. The block size will be randomized to six.

The treatment allocation codes will be enclosed in sequentially numbered opaque envelopes by an independent researcher; the research assistant will pick the envelope and give it to the acupuncturist when the participants have finished all baseline assessments and immediately begin the first acupuncture treatment. The principal investigator (PI), co-investigators (Co-Is), and independent outcome assessors will be blinded to the treatment assignment. Only the acupuncturists will know to which groups the participants belong.

#### Blinding

Participants will be told that they will be randomly assigned to either acupuncture treatment or acupuncture-like simulation treatment and will be asked to wear an eye-patch when they receive treatment. Only the acupuncturists will know the group assignments. The participants and other researchers (including the data analysts, outcome assessors, and statisticians) will be blinded to group allocation. In order to ensure the successful implementation of the blinding method, all researchers will be trained before the trial begins.

#### Blinding success assessment

After the final treatment session, the success of blinding will be tested by asking the participants the following question “When you volunteered for the study, you were informed that you had an equal chance of receiving traditional acupuncture or acupuncture-like simulation treatment. Our study is finished now, which style acupuncture do you think you are received?” Three choices will be provided for participants: acupuncture treatment group; acupuncture-like simulation treatment group; and uncertain group. If participants choose “uncertain,” we will ask the reasons why they have made that assumption.

#### Safety and undesirable effects

Patients will be advised in the event of any undesirable effect after the application of acupuncture that they should discontinue acupuncture and contact their doctors; the doctor will diagnose and treat the adverse reactions. They will also be asked to contact the researcher who will have completed an undesirable effect form as well as the undesirable effect record log. Adverse events (AE) such as fainting, allergies, and pain may occur in acupuncture clinical trials [30]. If the participant faints, the needle will be immediately withdrawn. The patient will be returned to a supine position and given warm water or sugar water. The patient will be allowed to rest until they have made a full recovery. In the case of pain and allergies, the needle should be taken out immediately and treated according to the specific circumstances.

Any AE (include any discomfort, symptoms, or diseases occurring during the trial) will be reported by the patients and doctors after acupuncture treatment. All details of AEs will be reported in the CRFs. At the end of the study, we will analyze the influence of all events.

#### Data management

Patient characteristics will be recorded in CRFs, which will be stored in the researcher’s work office, and codes and initials will be used instead of the participants’ information to protect the participants’ privacy. Quality of CRF completion will be monitored by the specified researcher. All the original RCT data will be entered into the ResMan Research Manager of the Clinical Trial Management Public Platform. The relevant users will be trained and the system will be tested before it is officially launched to ensure that the system meets the trial requirements. Only relevant personnel will receive the account number and password once the system is officially launched. The clinical supervisor will monitor the work of the clinical trial center at least once a month.

### Statistical analysis

All data will be analyzed by intention-to-treat (ITT), including data from any participants who have dropped out of the RCT during the trial. All data will be entered twice by two different researchers to ensure the accuracy. If the data are found to be uncertain, the data supervisor will notify the researcher to respond with a data question form. If necessary, the statistician will send a data question form to the researcher and the researcher’s answer should be filled in the form. The question form is returned to the statistician by the inspector. The statistical analysis of data will be carried out by SPSS 23.0 software. Descriptive characteristics of baseline statistics will be reported as the mean ± standard deviation or median.

To analyze the primary outcome, the change of PSQI scores between baseline and week 8 will be calculated; comparisons between acupuncture group and sham-acupuncture group will be made using the Student’s t test. The rank-sum test is used for ranked data, while the 휒^2^ test is adopted to analyze categorical data. For other secondary outcomes, ISI, SAS, SDS, and MenQoL and Actigraphy assessments (TST and SA) between the two groups will be compared with the Student’s t test or the Wilcoxon rank-sum test. Analysis of secondary outcomes are considered exploratory; they were not part of the power calculations. All tests are two-tailed. *P* values ≤ 0.05 will be considered statistically significant.

### Monitoring

To improve the quality of this RCT, the whole process will be conducted under the supervision of a qualified clinical trial expert and be carried out by Shanghai Municipal Hospital of Traditional Chinese Medicine. The Clinical Research Center of Drugs of the Shanghai University of Traditional Chinese Medicine will provide data monitoring with access to any interim results and will make the final decision to terminate the trial if necessary. It also identify problems in the project, if any; the center makes the decisions to change the details of this protocol and announce the persons conducting the trial by written notice after approval by the application ethics committee. In addition, a qualified clinical trial expert will be invited to monitor this study and the PI will take full responsibility and will make the final decision.

## Discussion

Acupuncture treatment has been practiced in China for more than two millennia [31] and in recent years has been widely used in Western countries [32]. Recent studies have found that acupuncture can be used to relieve vasomotor symptoms during the MT [23], but have not yet established the effects of acupuncture on insomnia during the MT. This is a valuable clinical RCT aimed at treating insomnia in menopausal women. We use acupuncture and sham acupuncture [33] to evaluate the efficacy of acupuncture on improving sleep quality. In addition, we use a strictly RCT, utilizing the control group method to reduce test bias and ensure the reliability of the RCT.

The improvement on previous trials offered in this RCT is the method of point selection. Individualized treatment upon syndrome differentiation is the basis of TCM theory [23]; the selection of points is a question for the world acupuncture researchers [34]. To give some evidence for the effects of treatment upon syndrome differentiation, we set eight points as main points and add two acupoints based on individual differences in the syndromes. Another advantage is the use of Actigraphy. This wrist-worn sleep monitor can provide an objective assessment for the treatment effect [35]. This is very useful for us to compare and evaluate the change of patients’ sleep quality before and after treatment.

Another innovation in this trial is the design of the treatment frequency. In the first month, we will give full dose (three times per week) treatment. After the first month, we will decrease the frequency of the acupuncture treatment. This will be beneficial for us to observe the acupuncture therapeutic effect and to achieve the purpose of maintaining the minimum therapeutic dose in the long term.

However, this RCT still faces some challenges. First, the single-blinded method. For clinical trials of acupuncture, it is inevitable that acupuncturists know about the acupuncture treatment or sham acupuncture treatment. All participants will be asked to wear an eye-patch and will be arranged in a separate quiet space during the treatment. Second, the application of the sham acupuncture method. The acupuncturists will receive several sessions of strict training before the study begins to ensure the proper sham acupuncture techniques. Finally, the challenge of participant compliance. We will provide patient outreach over the phone to arrange the treatment time reasonably and to improve the attendance rate of the patients. The researchers will conduct follow-up interviews by phone to record patient outcomes.

To achieve our clinical goals, we will strive to standardize every step of the study, including acupoints selection, acupuncture operation, needle apparatus, and therapists’ clinical experience. We expect that this trial will provide strong positive evidence for acupuncture treatment of sleep disorders in the perimenopausal period.

## Trial status

The first investigators’ meeting took place on 31 August 2018. The RCT is in preparation now and will launch in November 2018. Recruitment is expected to end in late 2020.

## Abbreviations

CRF: Case report form
EX-HN: Extra acupoints on head
GV: Governor vessel
HT: Heart meridian of hand Shaoyin
HT: Hormone therapy
ISI: Insomnia Severity Index
ITT: Intention-to-treat
KI: Kidney meridian of foot Shaoyin
MenQOL: Menopause Quality of Life
MT: Menopausal transition
PSQI: Pittsburgh Sleep Quality Index
RN: Ren meridians
SAS: Self-Rating Anxiety Scale
SDS: Self-Rating Depression Scale
SP: Spleen meridian of foot Taiyin
TCM: Traditional Chinese medicine
TST: Total sleep time
